# Death of Dr. W. G. A. Bonwill

**Published:** 1899-10

**Authors:** 


### DEATH OF DR. W. G. A. BONWILL.

It is with profound regret that we have to announce the death of this distinguished worker in our profession, which took place September 24, 1899, after an illness of some seven weeks.

Further notice of his life-work must be deferred until our next issue.

The dental profession loses one of its most active and original workers, and the writer a friend of many years.

